# Biology of archaea from a novel family *Cuniculiplasmataceae* (*Thermoplasmata*) ubiquitous in hyperacidic environments

**DOI:** 10.1038/srep39034

**Published:** 2016-12-14

**Authors:** Olga V. Golyshina, Ilya V. Kublanov, Hai Tran, Alexei A. Korzhenkov, Heinrich Lünsdorf, Taras Y. Nechitaylo, Sergey N. Gavrilov, Stepan V. Toshchakov, Peter N. Golyshin

**Affiliations:** 1 School of Biological Sciences, Bangor University, Deiniol Rd, Bangor, LL57 2UW, UK; 2 Winogradsky Institute of Microbiology, Research Center for Biotechnology Russian Academy of Sciences, Prospect 60-Letiya Oktyabrya 7/2, Moscow, 117312, Russia; 3 Immanuel Kant Baltic Federal University, 236040 Kaliningrad, Russia; 4 Central Unit of Microscopy, Helmholtz Centre for Infection Research, Inhoffenstrasse 7, Braunschweig, 38124, Germany; 5 Insect Symbiosis Research Group, Max Planck Institute for Chemical Ecology, Hans-Knöll-Strasse 8, Jena, 07745, Germany

## Abstract

The order *Thermoplasmatales* (*Euryarchaeota*) is represented by the most acidophilic organisms known so far that are poorly amenable to cultivation. Earlier culture-independent studies in Iron Mountain (California) pointed at an abundant archaeal group, dubbed ‘G-plasma’. We examined the genomes and physiology of two cultured representatives of a Family *Cuniculiplasmataceae,* recently isolated from acidic (pH 1–1.5) sites in Spain and UK that are 16S rRNA gene sequence-identical with ‘G-plasma’. Organisms had largest genomes among *Thermoplasmatales* (1.87–1.94 Mbp), that shared 98.7–98.8% average nucleotide identities between themselves and ‘G-plasma’ and exhibited a high genome conservation even within their genomic islands, despite their remote geographical localisations. Facultatively anaerobic heterotrophs, they possess an ancestral form of A-type terminal oxygen reductase from a distinct parental clade. The lack of complete pathways for biosynthesis of histidine, valine, leucine, isoleucine, lysine and proline pre-determines the reliance on external sources of amino acids and hence the lifestyle of these organisms as scavengers of proteinaceous compounds from surrounding microbial community members. In contrast to earlier metagenomics-based assumptions, isolates were S-layer-deficient, non-motile, non-methylotrophic and devoid of iron-oxidation despite the abundance of methylotrophy substrates and ferrous iron *in situ*, which underlines the essentiality of experimental validation of bioinformatic predictions.

Acidic environments are widely distributed across the globe and are represented by natural (e.g. volcanic or geothermally heated), or man-made (mines or acid mine drainage (AMD)) sites, with a constantly low pH^1^. Microbial communities inhabiting such niches were considered to be of a relatively low complexity^2^, however, recent OMICS studies pointed at a greater variety of yet uncultured prokaryotes^1^. Due to the low numbers of cultured microorganisms that may serve as a functional reference, their physiological features and hence the roles in the environment largely remain at the level of *in silico* predictions from metagenomic data. In that context, while a certain success has been achieved in isolation of new bacterial taxa from these specific environments^3^, only a handful of cultured, taxonomically described and physiologically studied archaeal representatives have been obtained^3^. Recent data based on genomes assembled from metagenomes documented a number of archaeal clades mostly affiliated with the order *Thermoplasmatales,* phylum *Euryarchaeota*^4^. Among archaeal populations from the above order, cultured members of *Ferroplasmaceae* together with yet uncultured archaea from the so-called ‘alphabet plasmas’ were the most abundant and hence suggested to play important roles in carbon cycling in the environment^5^. Initially identified in 16S rRNA gene clone libraries from Iron Mountain^6^, these archaea have later been found in a number of acidic environments of different temperature regimes^1^. Their presence in iron-rich environments have quite logically promoted discussions on their iron oxidation potential. Apart from the iron oxidation experimentally confirmed only in cultured members of *Ferroplasmaceae*^7^,^8^, other *Thermoplasmatales* were described as facultatively anaerobic heterotrophs^9^. Their appearance in biofilms alongside with chemolithoautotrophs suggests that the metabolism of this group may be depended on organic compounds (sugar polymers/oligomers, peptides, lipids or carbohydrate monomers) derived from primary producing organisms^10^. “Alphabet plasmas” were furthermore predicted to oxidise carbon monoxide and utilise methylated compounds^4^. However, the dearth of experimental evidence has largely limited our entire current understanding on metabolism, physiology, and environmental roles of these archaeal lineages.

One of important members of *Thermoplasmatales* in AMD systems from Iron Mountain (California) was a group of organisms dubbed ‘G-plasma’, which was so abundant, that the genomes of some of the representatives were almost fully assembled^2^,^4^. These organisms were the third-abundant community members (following *Leptospirillum* spp. “Group II and III”) and contributed up to 22% of total community proteome^11^. Elsewhere, ‘G-plasma’ contributed approx. 15% of total metagenomic reads in this environment^12^. However, despite their abundance and ubiquity these organisms escaped the cultivation until very recently, when first representatives were isolated from Cantareras AMD site (Spain) and Parys Mountain/Mynydd Parys (Anglesey, UK) and described as representatives of a novel family, *Cuniculiplasmataceae,* new genus and species *Cuniculiplasma divulgatum* within the order *Thermoplasmatales*^13^. The 16S rRNA gene sequences of isolates PM4 and S5 were identical to those from ‘G-plasma’ cluster from metagenomic data analysed at Richmond mine at Iron Mountain, USA^2^,^4^, terrestrial acidic springs in Japan^14^, high-temperature fumarole and acidic biofilm from Mexico (GenBank Acc Nrs. JX997948, AB6000334 and KJ907759), Frasassi hydrogen sulphide-rich cave system, Italy^12^ and from AMD system, Los Rueldos, Spain^1^,^10^ and other low pH systems. Altogether, these documentations point at the ubiquity of *Cuniculiplasmataceae*-related organisms in acidic systems and volcanic areas (Fig. 1, Supplementary Table S1).

New isolates provided a great opportunity to perform for a first time the comparative genomic analysis of very closely related members of *Thermoplasmata* from very distant geographic locations, to analyse their physiology and functions related to the environment in the context of the earlier genomic predictions, and, finally, to analyse their evolutionary relationships with other clades within the class *Thermoplasmata*, which harbours organisms known as acidophilic ‘champions’^9^,^15^.

## Results

### Physiological traits: *in silico* predictions in ‘G-plasma’ vs experimental data

#### Iron oxidation

Despite earlier suggestions of iron-oxidising capabilities based on the occurrence of rusticyanin/sulfocyanin-encoding gene homologs^4^, no iron oxidation was confirmed with ferrous sulfate and pyrite in either *C. divulgatum* isolate. Noteworthy, the presence of genes for rusticyanin/sulfocyanin homologs might not necessarily be connected with the iron oxidation in archaea of the order *Thermoplasmatales,* e.g. *Picrophilus torridus* does not oxidise ferrous iron despite the presence of sulfocyanin. It was suggested^16^ that this respiratory complex in *P. torridus* is situated on a genomic island, which seems also to be the case for rusticyanin/sulfocyanin genes acquired by a lateral transfer in *C. divulgatum* S5 (s. below) and *F. acidiphilum* (Golyshina *et al*., in preparation).

#### Archaeal flagella and pili

**Figure 2:**
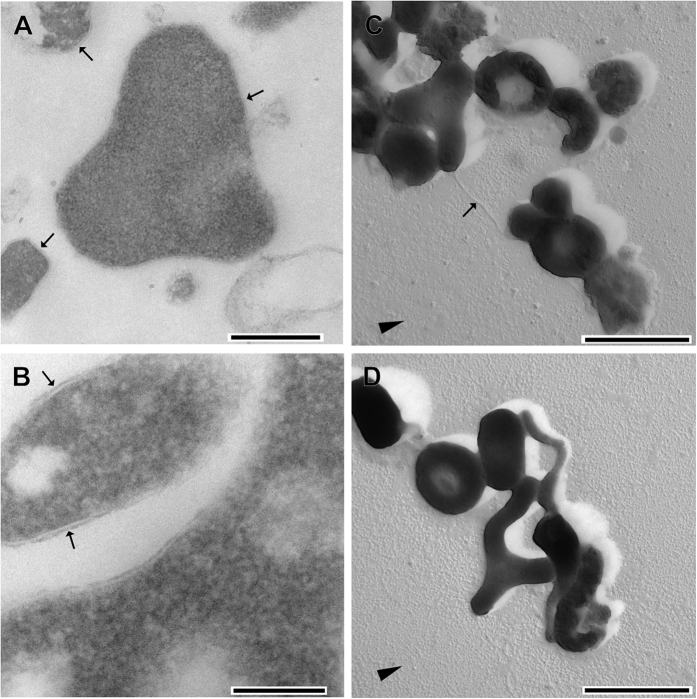
Electron micrographs of *Cuniculiplasma divulgatum* showing monolayer membranes and absence of the S-layer (**A,B**), pilus (**C**), arrow) and pleomorphism of cells. Scale bars: 500 nm (**A**), 200 nm (**B**), 1 μm (**C,D**). Ultrathin sections (**A,B**) and Pt-C shadow castings (**C,D**). Figure shows cells of the strain PM4 (**B,C** and **D**) and S5 (**A**). Arrowheads in **C** and **D** indicate the direction of shadow cast, arrows in **A** and **B** point to the cytoplasmic membrane.

In ‘G-plasma’, the full operon encoding FlaBCDEFGHIJ with individual proteins being homologous to those from *Methanococcus voltae* and *Halobacterium salinarum* has been reported earlier^4^, however, corresponding loci have not been detected in either genome of *Cuniculiplasma divulgatum*. Our analysis suggested that *M. voltae* and *H. salinarum* flagellar proteins do not have significant (e-values <0.01 and query coverage > 50%) BLAST hits in ‘G-plasma’ *in silico* translated proteome. Electron microscopy of *C. divulgatum* grown under optimal condtions did not provide evidence for an archaellum, but occasionally showed the presence of distinct pili^13^, (s. also Fig. 2C). This feature is also reflected in the genomic data of both isolates of *Cuniculiplasma*, as discussed further (s. subsection ‘*Secretion system and motility’*).

Regarding the S-layer prediction in ‘G-plasma’, corresponding genes to be linked with S-layer formation^4^ were also found in both *Cuniculiplasma* genomes. These genes annotated in ‘G-plasma’ to code for “S-layer protein *P. torridus*”^4^ (and annotated as a surface protein in *P. torridus* itself), are affiliated with COG3391, arCOG0652 and arCOG2560 that have five homologs in each *Cuniculiplasma divulgatum* genome). Additionally, genes encoding oligosaccharyltransferase AglB in ‘G-plasma’ are present in both genomes of *Cuniculiplasma* strains, as well. However, as revealed by electron microscopy, the cells of strains S5 and PM4 were only surrounded by cytoplasmic membranes and lacked distinct (predicted in ‘G-plasma‘) S-layers (Fig. 2A,B). An S-layer should provide a certain rigidity to cells and its absence is consistent with the characteristic pleomorphism in *C. divulgatum,* as exemplified in Fig. 2C,D. Apparently, within the order *Thermoplasmatales,* the cell wall-deficient members clearly outnumber S-layer-exhibiting organisms, which are represented only by *Picrophius* spp.^9^. This feature is also reflected in the genomic data of two strains, as discussed further.

#### Methylotrophy

In the growth experiments performed with both strains of *C. divulgatum* with a range of methylated compounds^13^ we were not able to confirm the methylotrophy earlier predicted in ‘G-plasma’^4^. In this regard, the genes predicted to be present in ‘G-plasma’, namely methenyl tetrahydrofolate cyclohydrolase and formyl-tetrahydrofolate synthetase have also been found in both *Cuniculiplasma* genomes. However, the gene encoding ‘methanol dehydrogenase’ in ‘G-plasma’ has not been confirmed in *Cuniculiplasma*. Furthermore, the protein referred as such in ‘G-plasma’ itself had a low amino acid sequence identity (>26%) to alcohol dehydrogeneases of unknown substrate specificity and was equally (dis)similar with maleylacetate reductases. The very homolog was found in the S5 genome, but not in PM4. Among tested substrates, e.g. methylamines, could not be utilised by *Cuniculiplasma* isolates since no genes for methylamine dehydrogenase or dimethylamine and trimethylamine dehydrogenase were found. Whatever the case, methylotrophy was not experimentally confirmed in any *Thermoplasmatales,* even though the methanol is a common product of organic matter degradation and may be available in studied environments.

### Genome analysis of *Cuniculiplasmataceae*

#### Genome statistics

The genomes of *C. divulgatum* strains (Table 1) are larger as compared to the relatives from *Thermoplasma* spp (1.58 Mbp for *T. volcanium* and 1.56 Mbp for *T. acidophilum*) and *Picrophilus torridus* (1.55 Mbp), being within the common range to archaea of the family *Ferroplasmaceae* (1.94 Mbp for *“Ferroplasma acidarmanus”,* 1.75–1.78 Mb for *Acidiplasma aeolicum* and 1.74 Mbp for *A. cupricumulans*). Low G+C contents of genomic DNA of strains S5 and PM4 are rather typical for *Thermoplasmatales*^17^.

#### Genome comparisons

The three genomes exhibited a high average nucleotide identity (ANI)^18^ and average amino acid identity (AAI)^19^, which also support similar physiological patterns in both isolates: strains S5 and PM4 had 98.8% ANI, while ANI of both isolates with ‘G-plasma’ genome were about 98.7 and 98.4%, respectively, pointing at their similar evolutionary trajectories despite transcontinental localisation of their niches and highly complementary microbial structure and gene pools in AMD settings^1^ (also s. Fig. S1 for the AAI data).

**Figure 3:**
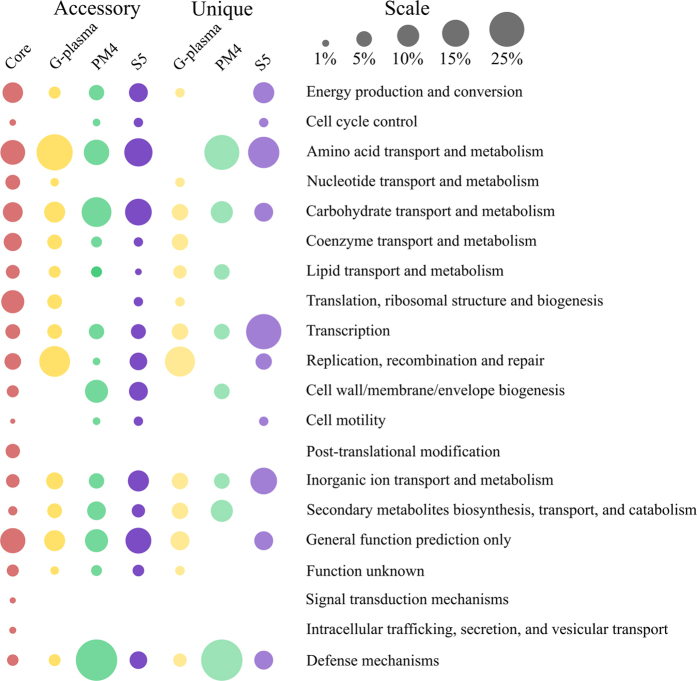
Distribution of arCOG Functional Categories within core, accessory and strain-specific (unique) proteomes of *C. divulgatum* S5 and PM4 and ‘G-plasma’. Circle area is proportional to the fraction of corresponding arCOG FC to the total number of arCOG hits in every group of proteins. Core group corresponds to proteins found and all three genomes, accessory group includes proteins found in at least two genomes and unique group consists of strain-specific proteins.

**Figure 4:**
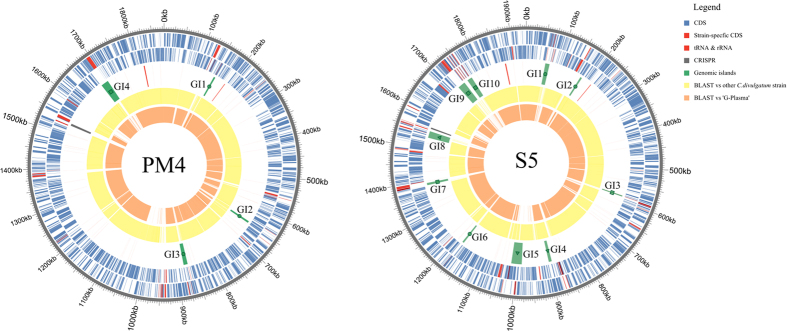
Genomic islands (GIs) in *C. divulgatum* strains PM4 and S5. Rings from outside to inside: genomic coordinates (grey colour); plus-strand CDS (blue) and RNA (red);); minus-strand CDS (blue) and RNA (red); strain-specific CDS (red) and genomic islands (green); blastn hits with e-value cutoff 10^−5^ vs other *C. divulgatum* isolate (yellow); blastn hits with e-value cutoff 10^−5^ vs ‘G-plasma’ (orange). Function of GIs is marked by small figures: ‘defense’ islands – squares, ‘transporter’ islands – triangles, islands of non-specific function – circles.

The core *in silico* proteome of *C. divulgatum* strains and ‘G-plasma’ is represented by 1174 protein groups. 111 protein clusters were identified as exclusively distributed among PM4 and S5 strains, 13 among PM4 and ‘G-plasma’ and 27 among S5 and ‘G-plasma’ (Figs 3 and 4 and Fig. S1, Supplementary Table S2). 79, 52 and 114 unique single-copy genes and 1, 1 and 10 strain-specific paralogue clusters were identified for S5, PM4 and ‘G-plasma’ respectively. Analysis of their distribution across the chromosomes revealed that most of them are highly clustered (Fig. 4), supporting the hypothesis that LGT (lateral gene transfer) is an important driving force in evolution of AMD-related microorganisms^20^, however with very similar patterns of foreign DNA integration in the genomes of recipients.

#### Lateral gene transfer (LGT), genomic islands (GIs) and defence systems

Analysis of arCOG distribution within variable and core parts of *Cuniculiplasma*-related *in silico* proteomes revealed a significant enrichment in “Defense mechanisms” group in PM4 strain. This observation together with the fact that PM4 possesses 92 non-redundant CRISPR spacers as opposed to 52 in S5 strain and only 10 in ‘G-plasma’ give an opportunity to speculate that Parys Mountain/Mynydd Parys mine is characterised by much higher viral load than other investigated acid mine habitats^21^. In turn, unique and accessory part of ‘G-plasma’ genome characterised by the lowest proportion of ‘defence mechanisms’ is highly enriched with ‘replication, recombination and repair’ proteins including integrases, transposases and recombinases pointing on higher level of genome mobility in ‘G-plasma’ (Fig. 3). Another point related to arCOG distribution is the prevailing comparative number of unique strain-specific proteins in S5 for categories energy production and conversion, cell cycle control, transcription, inorganic ions transport and metabolism (Fig. 3).

The strain S5 harboured ten GIs in its genome, whereas its counterpart from Parys Mt/Mynydd Parys only four (Fig. 4(A,B) and Supplementary Table S2). As expected, numerous insertion sequences elements (IS), integrases and transposases from different families (IS3, IS5, IS6, IS66, IS256, IS200/605, IS110, IS1634) were associated with the GIs, as well as tRNAs reflecting the commonality of tRNA co-occurrence in genomic islands^22^. The G+C molar content in predicted GIs varied within the range 37.7–43.2%, i.e. marginally higher than average values in PM4 and S5 genomes (Supplementary Table S3), which may be a result of old integration events and consequent DNA amelioration in GIs making GC similar to that in the core genomes. Notably, a slight difference between G+C-content in genomes of S5 and PM4 strains (37.16% in PM4 vs 37.30 in S5) is determined by the presence of six additional GIs in the former isolate. Analysis of taxonomic affiliation of GIs revealed that almost all lateral transfers originated from other acidophilic euryarchaea. This observation implies the existence of a highly mobile gene pool in acidophilic *Archaea*, which determines rapid adaptations of *Thermoplasmatales* members to toxic concentration of heavy metals and to a high viral load.

Thus, some GIs could clearly be attributed to ‘defence’ islands, e.g. GI 3, GI 7 and GI 9–10 in S5 and GI 4 in PM4 due to the localisation therein of genes for restriction-modification and toxin-antitoxin systems. Others (e.g. GI 4, GI 5 and GI 8 from S5) were transport-, efflux-, metal- and oxidative stress response-related). GI 1 from the strain S5, which is absent in PM4, harboured an array of genes for site-specific recombinases, metal-transporting ATPases, multipass membrane proteins, metallochaperones, cupredoxin COX2 family proteins, heavy metal reductases, and rustycyanin/sulfocyanin homolog.

We have identified several toxin-antitoxin systems (TAS) -encoding genes, mostly associated with GIs in both isolates. The most abundant ones were represented by *vap*BC of the type II system: six clusters of corresponding ORFs in PM4, and seven in S5 and, besides three *vap*B toxin genes were found across chromosomes in both *Cuniculiplasma* isolates. In addition, three clusters of genes were found in PM4 and two such loci in S5 with corresponding MazE and MazF family proteins affiliated with COG2336/arCOG03943 and COG2337, respectively.

Furthermore, three and two *rel*EF loci were identified in PM4 and S5 genomes, correspondingly. Commonly, TAS are known to be stress response-connected and lateral gene transfer-related^23^,^24^, which is confirmed by the GI analysis. Notably, no TAS were previously reported in *Thermoplasmatales*^25^.

All genomes of *Cuniculiplasma* spp. showed the presence of Clustered Regularly Interspaced Short Palindromic Repeats (CRISPR)-Cas defence systems: in S5, we have identified the cluster of genes for Cas3, Csx17, Cas7, Cas5, Cas4/Cas1 and Cas2 with an adjacent CRISPR repeat region with 57 spacers. Interestingly, all proteins exhibited 100% polypeptide identity with counterparts from ‘G-plasma’ (apart from Cas4 and 1 which had psi-blast hits of about 54% identity with acidobacterial polypeptides).

ATDV01000019 contig of ‘G-plasma’ exhibited a remarkable similarity in gene arrangement (ADMU5_GPLC00019G0101-G0107) with the corresponding region in S5 (CIP_1636–1642) albeit with only 10 spacers of repeats found on the terminus of the contig ATDV01000011. According to^26^ systems from ‘G-plasma’ and S5 can be classified as Type I-C. The strain PM4, in contrast to the above, coded, in this order, for Cas6 endoribonuclease, Cas8b, Cas7, Cas5, Cas3, Cas4, Cas1 and Cas2, flanked by a repeats-spacers array of 92 spacers, suggesting its affiliation with the Type I-B system^26^. Interestingly, all sequences of Cas proteins were equally distant (28–57% sequence identity (Supplementary Table S4) with the proteins from ‘*F. acidarmanus*’ and other archaea and, to the same extent, with polypeptides from representatives of *Bacteria,* e.g*. ∂-Proteobacteria* or *Acidobacteria* (pointing at an unclear origin of corresponding gene clusters). Remarkably, very similar pseudogenes CPM_1008 and CSP5_0996 for Cas1 were detected in both isolates, in similar locations on chromosomes, within the same genomic context in the region severely affected by transposon integration and pseudogenisation. Analysis of CRISPR repeats in S5, PM4 and ‘G-plasma’ by blastn-short algorithm revealed no cross-matches of spacers between these three genomes. Nevertheless, eight of 92 PM4 repeats and four of 57 S5 spacers showed high (90–100%) identity with sequences of Richmond mine microbial and viral communities^21^,^27^, suggesting the existence of viruses common for these extreme acidic ecosystems. Interestingly, CRISPR array of PM4 contains two spacers with the significant level of similarity (83 and 96%) to marine metagenomic sequences (Supplementary Table S5). Despite a significantly high probability of false positive hits (e-values are 0.015 and 0.14, respectively), this finding might be speculated as relic genomic signatures of an ancient hydrothermal ecosystem which existed 480–360 my BP in the place of contemporary Parys Mountain site^28^.

From the analysis of GIs in *Cuniculiplasma* spp. two important facts become apparent. First, the co-occurrence of GIs and the majority of ‘unique’ genes (numbers in the outermost segments in Fig. S1 and green lines in Fig. 3). Most ‘unique’ genes had likely been acquired from organisms other than *Thermoplasmata* and had no hits above the e-value cut-off (0.005) either with ‘alphabet plasmas’ or with isolates from cultured/genome-sequenced *Thermoplasmata*, suggesting a high probability of lateral gene transfer also in the vicinity of GIs. Second, a remarkable similarity in gene arrangements was observed within some GIs in both strains and their positioning in both chromosomes, i.e. in ‘defence islands’ GI 9–10 of S5 and GI 4 of PM4 (homologous to ‘G-plasma’ contig ADMU5_GPLC00019G0004-G0013) (Supplementary Fig. S2(b) and Supplementary Table S6) and GI 2 of S5 and GI 1 and 2 from PM4 that were mostly composed by ORFs for hypothetical proteins conserved in both organisms (Supplementary Fig. S2(a)). Such conservation in gene arrangements in GIs is indicative for an important role these genes may play in metabolism in iron-rich environments and that they can relatively easily be transferred between organisms and remain in genomes due to the selective pressure, providing a competitive advantage, much like ‘catabolic transposons’ for xenobiotics or hydrocarbon metabolism^29^. This was the case in, e.g., three transposases-adjacent operons in strain S5 encoding metallochaperone and metalloreductases that showed high similarities with counterparts in all *Thermoplasmatales* type strains.

#### Secretion systems and motility

In the genomes of both strains PM4 and S5 no operon essential for archaella biogenesis (*fla*CDFGB)^30^ was found, and consistently, no archaella and no motility were observed by microscopy, despite earlier suggestions^4^,^13^. The strain PM4 exhibited filaments or pili-like cell surface structures^13^, according with the presence in genomes of genes for proteins of type IV pili biosynthesis. In accordance with the recent census of archaeal clusters of orthologous groups of proteins (arCOG) related with pili formation^31^, we have identified principal components in both genomes as follows. In S5, CSP5_0712 and CSP5_0715 encoded Type II secretion system ATPase subunits (FlaI, arCOG01817) forming a gene cluster with genes for CSP5_0713-14, encoding homologs of flagellar assembly proteins J2 and J1 (TadC, arCOG01808) and major pilins (FlaB/FlaF/PilA family, arCOG02423) coded by clustered CSP5_1254-1255 and stand-alone CSP5_0804 and CSP5_0881. The arrangement of two gene clusters harbouring six former gene loci resembled that in both genomes of “*Aciduliprofundum*” spp.^31^. In the strain PM4, corresponding loci were CPM_0710 and 0713 (secretion ATPases), CPM_0711-0712 (TadC-like proteins) and CPM_1256-57, 0800 and 0878 (major pilins), with the very same arrangement of gene clusters across the chromosome, as in the strain S5. Function of these surface formations could be various: surface adhesion, intercellular connection, DNA exchange or probably attachment to the substratum rather than the motility^32^. Both strains encode type IV secretion components: TraG/TraD/VirD4 family ATPases (arCOG04816) by CSP5_0791 and CPM_0795; membrane protein (arCOG05340), VirB4 component (arCOG04034), multipass protein (arCOG05369) and membrane protein (conserved in *Thermoplasmatales* only) with four latter encoded by gene clusters CSP5_1185-1189 and CPM_1190-93. Furthermore, both genomes encode Sec translocon components, preprotein translocase subunits SecYE and Sec61beta, signal peptide peptidase and signal recognition particle subunits and receptors. Another feature to be addressed here is the presence of Sec-independent Tat pathway genes for folded proteins’ secretion. Twin-arginine translocase subunits A and C are presented in PM4 and S5 genomes, which may be functional in an analogy with a Gram-positive bacterial Tat system, known to work without additional TatB protein^33^.

#### Peptidases, peptide/amino acids transporters

Consistently with the substrate preferences for proteinaceous compounds, each genome contained more than 50 various peptidases. Among those, eight were predicted to be secreted due to the presence of signal peptides. Five peptidases were most probably responsible for extracellular hydrolysis of proteins and peptides: three serine peptidases of S53 family and two thermopsins, aspartic peptidases of A5 family^34^. S53 family peptidases have 3D structures similar with other representatives of SB clan, their distant homologs, subtilases of S8 family, but differ in acidic pH optima for activity. Since all *Thermoplasmatales* are extremely acidophilic microorganisms, it is quite logic that S8 peptidases-coding genes were not found in their genomes, and were ‘replaced’ by S53 peptidases. A thermopsin, also characterised as an acidic endopeptidase^35^ is another reflection of adaptation of *Cuniculiplasma* spp. to extremely acidic conditions. The genomes of the strains S5 and PM4 encoded two almost identical thermopsins, however, one of S5 thermopsins lacked 130 amino acid on its N-terminus and hence lacking secretion system motifs. Genomic context analysis revealed the presence of various transporters in close vicinity of A5 peptidases of both strains and almost no transporters in S53 neighbourhood. Among transporters, surrounding thermopsins, the most probable amino acid and peptides importers were among the members of Major Facilitator Superfamily (MFS, 2.A.1), according to TCDB database^36^.

#### TCA

All genes, coding for TCA proteins were clearly identified in *Cuniculiplasma* genomes except 2-oxoglutarate dehydrogenase (EC 1.2.4.2) and fumarate reductase (EC 1.3.5.1). A 2-ketoacid dehydrogenase complex was found (CSP5_0253-0256 and CPM_0219-0222), however it was related to rather 2-oxoisocaproate dehydrogenase (EC 1.2.4.4) than to 2-oxoglutarate dehydrogenase (EC 1.2.4.2) or pyruvate dehydrogenase (1.2.4.1). Still, the conversion of 2-oxoglutarate to succinyl-CoA could be catalyzed by 2-oxoglutarate synthases (CSP5_0284-0285 and CPM_0255-0253) and CSP5_1378-1379/CPM_1377-1378). These ferredoxin-dependent enzymes are known to be highly sensitive to oxygen, thus, presumably being active during anaerobic growth of *C. divulgatum* or being highly stable to oxygen as it was shown for a homolog from *Mycobacterium tuberculosis*^37^. CSP5_1895 and CPM_1834 (COG1027) are homologous to several characterised class II aerobic fumarases (EC 4.2.1.2), however the phylogenetic analysis shows (Supplementary Fig. S3) their marginally closer relatedness with aspartases (EC 4.3.1.1) than with fumarases (yet with high AA identity/similarity values (38/57%) with the Class II fumarase from *Sulfolobus* sp.). Whatever the case, a possible absence of fumarase would imply incompleteness of the TCA cycle, however it would still be able to generate the proton motive force via the Complex II (succinate dehydrogenase CSP5_0486-0489 and CPM_0468-0451). As expected, glyoxylate bypass seems to be inoperative: isocitrate lyase was found, but not the malate synthase.

In the course of growth of *C. divulgatum* on peptides, the lack of recirculation of TCA metabolites due to its incompleteness can be compensated by their synthesis from amino acids. During potential sugars-driven growth, PEP can be converted to oxaloacetate in a reversible reaction (which is not favourable, but possible), catalysed by GTP-dependent phosphoenolpyruvate carboxykinase (CSP5_1337 and CPM_1336) while malate or oxaloacetate can be synthesized from pyruvate by a reverse reaction catalysed by malic enzyme (CSP5_0838, CPM_0835).

Despite the generation of the proton motive force at aerobic growth (complex II) on peptides or sugars (the latter was not confirmed experimentally in the current experimental setup) TCA cycle enzymes play a crucial role in anabolism during growth on peptides at both aerobic and anaerobic conditions. For example, the mentioned above GTP-dependent phosphoenolpyruvate carboxykinase and malic enzyme uses its metabolites for the first stages of gluconeogenesis: phosphoenolpyruvate and pyruvate synthesis, respectively.

#### NADH dehydrogenase

Both *Cuniculiplasma* genomes contain genes for four major respiratory complexes, with some unusual details, as specified below. A set of genes of the proton-translocating type I NADH-dehydrogenase (complex I) *nuoABCDHIJJKLMN* is encoded by CSP5_1737-1726 in the strain S5 and CPM_1708-1687 in the strain PM4, in the same order. Both genomes encode neither NuoG subunit, nor subunits NuoE or NuoF homologs essential to provide the catalytic site for NADH oxidation, which raises doubts in NADH-oxidizing activity of the complex I and its involvement in respiratory electron transfer chain in *C. divulgatum*. Alternative pathway of electron inflow into the respiratory chain could be provided by succinate dehydrogenase/fumarate reductase (Complex II), encoded by CSP5_0486-0489 in S5 and CPM_0458-0461 in PM4 genomes. It should be mentioned that none of *Thermoplasmatales* genomes available to date contain genes of NuoEF subunits, indicating possibly inherent feature of respiratory complex I in the organisms of this deep phylogenetic lineage and possible existence of other yet unknown alternative mechanisms of electron flow from NADH oxidation – in analogy to those proposed in aerobically respiring *Helicobacter pylori,* also lacking NuoEF subunits of the complex I^38^.

#### Quinol oxidising complex III and oxygen respiration

Quinol oxidising complex III in both *C. divulgatum* genomes is represented by clustered genes of Rieske Fe-S protein and cytochrome *b* subunit of a typical cytochrome *bc*_*1*_complex encoded by CSP5_1460-1459 in the S5 and CPM_1454-1453 in the PM4 strains. These clusters are located remotely with the genes of terminal respiratory reductases in both genomes. No genes of an alternative complex III have been detected in *C. divulgatum* genomes.

**Figure 5:**
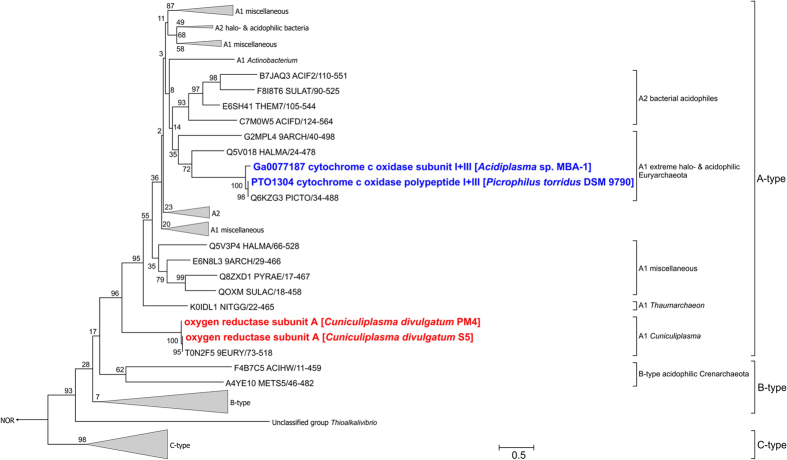
Maximum Likelihood phylogenetic tree of PF00115 polypeptides (COX1 family). Totally 112 sequences were used in analysis after 50% sequence identity filtering. The tree with the highest log likelihood (−68482.6023) is shown. The percentages of trees in which the associated taxa clustered together (bootstrap values, 1000 replicates) are shown next to the branches. The tree is drawn to scale, with branch lengths measured in the number of substitutions per site. All positions with less than 95% site coverage were eliminated. Unclassified group was first mentioned^42^. Nitric oxide reductases (NOR) were placed as an outgroup.

Terminal oxygen reductases are represented in both *C. divulgatum* strains by a typical cytochrome *bd* quinol oxidase (CSP5_0552-0553 and CPM_0524-0525) and a heme copper oxygen reductase (HCO) encoded in the clusters CSP5_1313-1312 and CPM_1312-1311. The first enzyme complex possesses a high affinity to oxygen and is usually involved in oxygen detoxification or respiration under microaerophilic conditions providing relatively low energy yield to the cell^39^. The heme-copper oxygen reductase (complex IV) is a typical terminal enzyme of aerobic respiratory electron transfer chain, coupling oxygen reduction to proton translocation at aerobic or microaerophilic conditions. Sequence analysis of catalytic subunits I of the heme-copper oxidases of *C. divulgatum* strains with a web-based classifying tool (http://evocell.org)^40^ clearly showed that both of them belong to the type A1 oxygen reductases possessing two proton translocating channels, and consequently, the highest proton pumping stoichiometry of 2H^+^ per one electron^41^,^42^,^43^. Our phylogenetic reconstruction of full-size CoxI available so far (Fig. 5) generally reproduced the recently reported topology of HCO phylogenetic trees and revealed that the A1-type heme-copper oxidases of *Cuniculiplasma* species form a distinct clade, most closely branching to B-type oxygen reductases and to the root of all the other A-type reductases. Interestingly, heme-copper oxygen reductases from other *Thermoplasmatales* (from *Acidiplasma* and *Picrophilus* species) are located on a distinct clade of A-type oxidases (Fig. 5).

Furthermore, both *C. divulgatum* strains lack genes for membrane-integral oxygen reductase subunits III and IV (either separately encoded or fused to the C-terminus of the catalytic subunit I), while those were found in *Acidiplasma* and *Picrophilus* genomes being fused with *coxI* genes. The subunits III and IV are regarded to be distinguishing features of A-type (SoxM) heme-copper oxygen reductases acquired during their evolution from less energetically effective and more ancient B-type enzymes^42^. The lack of these subunits in *C. divulgatum* together with the phylogenetic position of its CoxI proteins allows assuming that this organism possesses an ancestral form of all known A-type terminal oxygen reductases.

A crucial point for the activity of the heme-copper oxygen reductase is the pathway of electron transfer from the quinone pool or complex III. In *C. divulgatum* genomes, there are no genes of type I monoheme *c*-type cytochromes, providing the electron transfer from respiratory complexes III to terminal oxidases. Alternative pathway could be driven by blue-copper redox proteins (cupredoxins), as described in several acidophiles^44^. A homolog of such cupredoxins has been found to be involved in the respiratory chain of *Ferroplasma acidiphilum*^45^. As mentioned above, the gene encoding a cupredoxin rusticyanin was identified only in *C. divulgatum* strain S5 (CSP5_0076). The absence of both genes of type I cytotchrome *c* and rusticyanin/sulfocyanin does not allow predicting the electron transfer pathway between respiratory complexes III and IV in the strain PM4. The possibility still exists that the complex IV in strain PM4 possesses quinol oxidizing activity and, similarly to some other heme-copper oxidases, could directly accept electrons transferred from the complexes I and II via the quinone pool. In such a case, the complex III in strain PM4 would serve as an additional proton-translocating site, which is not directly involved in oxygen respiration and could transfer electrons to an extrinsic, yet unidentified acceptor. However, this assumption needs experimental evaluation.

All analysed genomes code for subunits K, E, C, F, A, B, D, H and I of V/A type H^+^-transporting ATP synthases, in this particular order (CSP5_0034-0042 and CPM_0034-0042).

Further central metabolic and protein folding pathways detailed in SI suggest *Cuniculiplasma* spp. largely share these with other *Thermoplasmata*.

### Comparison with other *Thermoplasmatales*

**Figure 6:**
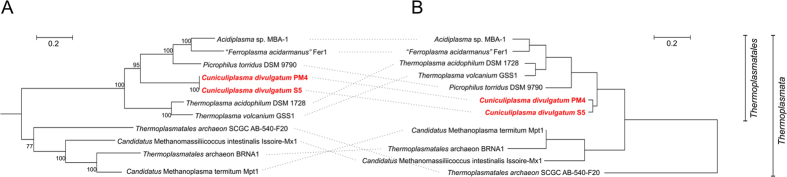
Phylogentic position of *Cuniculiplasma* spp. within *Thermoplasmata.* (**A**) Maximum Likelihood phylogenetic tree, based on concatenated sequences of 11 conservative ribosomal proteins of two *Cuniculiplasma* strains, nine *Thermoplasmata* representatives with the genomes, publically available in IMG and *Methanopyrus kandleri* AV19 as an outgroup (not shown on the tree). The proteins, involved into analysis were: COG0048, ribosomal protein S12; COG0049, ribosomal protein S7; COG0081, ribosomal protein L1; COG0197, ribosomal protein L16/L10AE; COG0200, ribosomal protein L15; COG0244, ribosomal protein L10; COG1631, ribosomal protein L44E; COG1890, ribosomal protein S3AE; COG2004, ribosomal protein S24E; COG2051, ribosomal protein S27E; COG2125, ribosomal protein S6E (S10). The tree with the highest log likelihood (−24738.5123) is shown. The percentages of trees in which the associated taxa clustered together (bootstrap values, 1000 replicates) are shown next at branching points. All positions with less than 95% site coverage were eliminated. There were a total of 1607 positions in the final dataset. The tree was constructed in MEGA6^53^. (**B**) IMG COG-based hierarchical clustering. The analysis was performed using IMG genomic annotations of two *Cuniculiplasma* strains and nine publically available *Thermoplasmata* representatives. Bars indicate the number of substitutions per site.

Phylogenomic analysis of *Thermoplasmata* based on concatenated amino acid sequences of 11 conservative ribosomal proteins of each representative of the phylum with a sequenced genome (Fig. 6A), indicates a slightly different tree topology than that suggested by 16S rRNA gene phylogenetic analysis^13^, likely due to this selection of particular molecular markers for phylogenetic reconstruction. On the other hand, IMG COG-based hierarchical clustering placed *Cuniculplasmataceae* representatives close to the root of the order *Thermoplasmatales* (Fig. 6B). This might be an indication that *Cuniculiplasma* spp. share more parental properties than other cultivated members of *Thermoplasmatales* and thus could be a good model for analysis of yet uncultivated members of the class *Thermoplasmata*.

In contrast to other *Thermoplasmatales*, the genome of *C. divulgatum* strain PM4 (but not S5) had no restriction-modification system Type I. Pyrimidine and purine conversion and utilization pathways, RNA processing and modification processes showed their incompleteness in *C. divulgatum*, in comparison to the rest of *Thermoplasmatales*. We also infer that amino acid biosynthesis category for other *Thermoplasmatales* (*T. acidophilum, P. torridus, and “F. acidarmanus”*) showed some discrepancies to *C. divulgatum*. Thus, *P. torridus* has been proposed to possess all pathways for the amino acid synthesis^16^. *“F. acidarmanus”* occurred to encode incomplete histidine, valine, leucine and isoleucine synthesis pathways^4^. The genomes inspection of *C. divulgatum* suggested, in addition to the above, incomplete pathways for lysine and proline, pointing at the organisms’ dependence on external peptides and hence suggesting their role in the environment as ‘scavengers’.

Incidentally, *C. divulgatum* and “*F. acidarmanus”* genomes, but not *T. acidophilum* or *P. torridus* encode proteins for capsular heptose metabolism and polyhydroxybutirate metabolism (with an exception of the gene encoding for acetoacetyl-CoA synthetase (EC 6.2.1.16 in *“F. acidarmanus”*).

The organisms have a weak potential for synthesis of polymeric storage compounds: both genomes for a similar folylpoly(gamma)glutamate synthase (CPM_0655 and CPM_1446). The PM4 also contains an inorganic polyphosphate/ATP-NAD kinase (CPM_0378) putatively active in energy gaining from environmental polyphosphate deposits. However, no cell inclusions were observed.

Formate dehydrogenase complex, involved into catabolism of C1 compounds, which is a common trait for *T. acidophilum* and *“F. acidarmanus”* has not been verified in either *Cuniciliplasma* genome. The gene coding for aquaporin Z (MIP superfamily) was found in both genomes of *C. divulgatum*, potentially contributing to the osmotic stress response and adaptive fitness, but absent in the genomes of other members of *Thermoplasmatales*. Another distinctive feature is the lack of molybdenum cofactor and coenzyme M biosynthesis in *C. divulgatum* genomes in contrary to other *Thermoplasmatales*. Finally, the lack of ATP-dependent DNA ligases in *C. divulgatum* genomes has been observed. The global analysis of distribution patterns of arCOGs in *Thermoplasmatales* is further detailed in SI and suggests *Cuniculiplasma* is a common member of the order.

## Discussion

Isolation of previously uncultured microorganisms from the environment remains one of the bottlenecks in microbiology hindering physiological and biochemical studies and demanding a resolution. It is especially important for archaea, the relatively recently discovered Domain, and which embraces a majority of difficult-to-culture organisms. The cultured diversity of archaea is dramatically low: according to the Euzeby LSPN online resource (http://www.bacterio.net/), only some 116 genera and 451 species with validly published names of archaea (of which 55–60% are haloarchaea-related organisms) *vs* some 2277 genera и 11940 species of cultured and described bacteria are known to-date. The acidophiles of the order *Thermoplasmatales* are a good example of this status of things, accounting for only six cultured genera published since 1970, despite numerous documentations on the presence of highly diverse *Thermoplasmatales*-like organisms in low-pH habitats worldwide. The present genomic analysis of new successfully cultured *Thermoplasmatales* members^13^ brought us closer to the understanding of functional diversity within this archaeal group. Interestingly, these archaea represent a unique case for *Thermoplasmatales*, when organisms from the same species and almost identical genomes from different geographic locations became cultured. Metabolically, *Cuniculiplasmataceae* resemble other *Thermoplasmatales* members, however certain discrepancies suggest some variety of their evolutionary trajectories. *Cuniculiplasma* spp. genomes encode the A1-type heme-copper oxidases forming a distinct clade at the root of A-type reductases and closely branching to the B-type oxygen reductases and are deficient in membrane-integral oxygen reductase subunits III and IV, suggesting that, in contrast with other *Thermoplasmatales,* they have a more ancient and less energetically efficient B-type enzymes. *Cuniculiplasma* spp. exhibit largest genomes among *Thermoplasmatales* seemingly at the expences of genetic loci for heavy metal resistance and defense systems. Scavenger type of nutrition was confirmed as a characteristic trait for *Cunicuiplasma* spp., which is reflected in their genomic blueprints and physiology, suggesting these organisms feed *in situ* on proteinaceous compounds derived from primary producing organisms. Based on the reconstructions of metagenomic data, the archaea related to this species previously supposed to be uncultured and associated to ’G-plasma cluster‘ are found in many acidic environments^1^,^6^. Certain features predicted from the metagenomic assembly “G-plasma” have not been confirmed highlighting the essentiality of cultivation efforts and experimental functional validation of genomic predictions. Almost identical genomes of the two European isolates and their North American sibling and strong conservation within their genomic islands, suggest a massive stabilizing selective pressure in similar acidic environments and/or significant fidelity of DNA repair systems assure their genome stability.

Isolation of reference strains and experimental validation of genomic predictions for this archaeal group should be considered in the future as tasks of a highest priority.

## Methods

### Sampling, and culturing, DNA isolation and sequencing

Samples from acidic streamers for isolation were taken in March-April of 2011 from Cantareras (Spain) and Parys Mountain/Mynydd Parys (UK) copper-containing sulfidic ores. Both cultures were grown in AB Medium, pH 1–1.2, as described previously^13^. DNA was isolated by GNOME DNA Isolation Kit (MP Biomedicals).

### Genome sequencing and analysis protocol

The genomes were sequenced at Fidelity Systems, Inc. (Gaithersburg, MD) using Illumina HiSeq 2000 platform, combining short paired-end libraries of 400 bp and long mate-paired 3,600 bp inserts with an average read length of 100 bases using manufacturer protocols with the only modification that for the PCR amplification of the genome library the TopoTaq DNA polymerase was used^46^. Initially, Velvet v. 1.2.10 was used to assemble the contigs^47^. Scaffolding, filling the gaps, and repeat resolution were performed using the Phred, Phrap, Consed software package^48^ and in-house software of Fidelity Systems. The error rate quality score of the completed genome sequences was of Phred 50. The final assemblies provided 564- and 561-fold coverages for strain S5 and PM4, correspondingly. The genome annotation was done at Fidelity Systems Ltd. using FgenesB 2.0 (SoftBerry, Inc., NY) followed by manual curation. The Rfam 11.0 database (http://rfam.sanger.ac.uk)^49^ and Infernal 1.0.2 (http://infernal.janelia.org)^50^ were used for annotation of RNA genes.

For analysis of shared and unique proteins all *in silico* translated genes were filtered by a length of 150 amino acids to exclude false predictions from the analysis. Resulting proteins were subjected to ‘all vs all’ alignment with blastp algorithm^51^ and e-value cut-off of 10^−5^. Resulting blast table was used as an input for OrthoMCL analysis with grain value of 2.5.

Assignment of predicted CDS to the archaeal clusters of orthologous groups (arCOGs) was made using blastp against the latest version of arCOG database^52^ with maximal e-value of 10^−5^. blastp hits were filtered to have minimum alignment length more than 50% of query and subject sequences length. arCOG was assigned to a protein if the hits to at least 3 different genera were registered.

Phylogenetic analyses were performed in MEGA 6^53^ using Maximum likelihood method and bootstrap confidence test. Sequence alignments were performed in MAFFT v. 7^54^.

Metagenome data search was performed through the following databases: MG-RAST^55^, IMG-M-ER databases^56^ and SRA archive^57^. Metagenome sequencing projects related to acidic environment were identified using keywords “acid” “mine” “drainage” “copper” and its combination. *Cuniculiplasma* related sequences were detected using blastn algorithm. Sequences with identity >95% were considered positive hits for MG-RAST and IMG-M-ER, while for NCBI SRA sequences identity cut-off was set to 99%. CRISPR repeat sequences were analysed locally using blastn tool of NCBI blast 2.4.0+ package against NCBI nt/nr, env_nt and htgs databases, PM4, S5 and ‘G-plasma’ genomes and against metagenomes acidic environments found in IMG-M database (Gp0051182, Gp0097388, Gp0097859, Gp0097858, Gp0053344 and Gp0053343). Parameters were as follows: word size: 7, match score: 1, mismatch penalty: −1, gap open penalty: 10, gap extension penalty: 2, percentage of query covered: 90, percentage identical bases: 90.

Genomic islands (GIs) in *C. divulgatum* were inspected using Island Viewer 3^58^.

## Additional Information

**How to cite this article**: Golyshina, O. V. *et al*. Biology of archaea from a novel family *Cuniculiplasmataceae* (*Thermoplasmata*) ubiquitous in hyperacidic environments. *Sci. Rep.* **6**, 39034; doi: 10.1038/srep39034 (2016).

**Publisher's note:** Springer Nature remains neutral with regard to jurisdictional claims in published maps and institutional affiliations.

## Supplementary Material

Supplementary Information

Supplementary Table S5

Supplementary Table S6

## Figures and Tables

**Figure 1:**
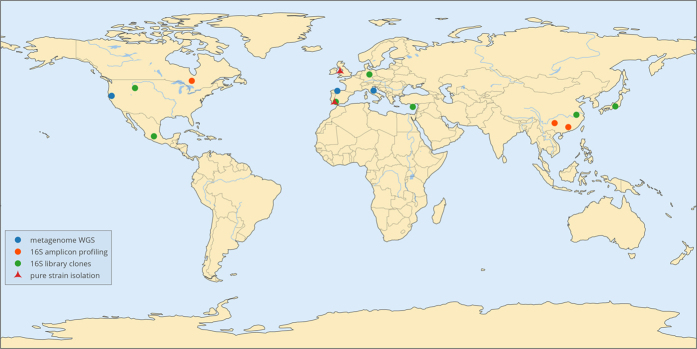
Worldwide distribution of *Cuniculiplasma*-related archaea. Map was created using Plotly online package (https://plot.ly/) using geographical coordinates, retrieved from metadata of database entries.

**Table 1:** Overview of general genomic features of *Cuniculiplasma divulgatum*, strains PM4 and S5 and ‘G-plasma’.

	Strain PM4	Strain S5	G-plasma*
Number of bases	1878916	1938699	1827255
Number of chromosome contigs	1	1	22
introns	1	5	ND
GC mol %	37.16	37.3	38.9
Coding density, %	87.1	87.4	88.5
Genes	1948	2016	1923
tRNA	46	46	48

^*^“G-plasma” genome stats may be affected by the application of a different annotation pipeline and the fact that it was assembled from metagenomic reads^4^ as opposed to the genomic assembly of pure cultures of strains S5 and PM4. ND, not determined.
