# Supplementary material for: Biology of archaea from a novel family Cuniculiplasmataceae (Thermoplasmata) ubiquitous in hyperacidic environments
Source: Sci Rep. 2016 Dec 14;6:39034. doi: 10.1038/srep39034 (PMC5155288; doi:10.1038/srep39034)
Supplement: Supplementary Information [file srep39034-s1.pdf]

Supplementary Information to the Manuscript

Biology of archaea from a novel family *Cuniculiplasmataceae* (*Thermoplasmata)* ubiquitous in hyperacidic environments

Golyshina O. V., Kublanov I. V., Tran H., Korzhenkov A. A., Lünsdorf H., Nechitaylo T. Y., Gavrilov S. N., Toshchakov S. V., Golyshin P. N.

Content

Supplementary text:

Central carbohydrate metabolic pathways *Pg 2*

Resistance to antibiotics and toxic compounds *Pg 3*

Oxidative stress *Pg 4*

Protein folding

Distribution patterns of arCOGs in *Thermoplasmatales* *Pg 5*

Supplementary Figures *Pg 6*

Supplementary Tables *Pg 10*

SI References *Pg 17*

Central carbohydrate metabolic pathways

Genomic mining predicted di- and oligosaccharides metabolism (maltose and maltodextrin utilization and trehalose biosynthesis), fermentation (acetyl-CoA fermentation to butyrate and butanol biosynthesis), monosaccharides metabolism (D-gluconate and ketogluconates metabolism, D-ribose utilization and mannose metabolism), one-carbon metabolism by tetrahydropterines, organic acids metabolism (glycerate, lactate and methylcitrate cycle) and glycogen metabolism to be credible in S5 and PM4 genomes. However, confirmed activities include peptides fermentation only.

Of modified Embden-Meyerhoff (EM) pathway both cuniculiplasmas have (in S5 numeration) ATP hexokinase (CSP5_0590), glucose/mannose-6-phosphate isomerase (CSP5_0657), fructose 1,6 biphosphatase (CSP5_1384), phosphorylating NADP+ Glyceraldehyde-3-phosphate dehydrogenase GAPDH (CSP5_0332) and phosphoglycerate kinase PGK (CSP5_0458), phosphoglycerate mutase (CSP5_0397), enolase (CSP5_1291) and pyruvate kinase (CSP5_1483). Neither ATP 6-phosphofructokinase, nor PPi or ADP 6-phosphofructokinases as well as classical fructose-bisphosphate aldolases classes 1 & 2 were found indicating the EM pathway working exclusively in synthetic direction with the key enzyme fructose 1,6 biphosphatase (CSP5_1384). On the other hand, few steps of glycolytic direction seem to be operational providing metabolites for Entner-Doudoroff (ED) and pentosophosphate (5P) pathways (see below).

According to[1,2,3] *Thermoplasmatales* use only mdED for glucose utilization. The following genes of the modified Entner-Doudoroff pathway enzymes were found in both genomes: glucose/galactose dehydrogenase GDH (CSP5_0708), gluconolactonase (CSP5_0139), gluconoate/galactonate dehydrogenase GAD (CSP5_2016), 2-dehydro-3-deoxy-gluconate/galactonate aldolase KDGA (CSP5_1346), D-glyceraldehyde

dehydrogenase GADH (CSP5_1304) and glycerate kinase (CSP5_1111). Generated glycerate-2 phosphate further converted to pyruvate by enolase and pyruvate kinase. Since no genes, coding enzymes, catalyzing phosphrilation of D-gluconate/galactonate (gluconokinase) or 2-dehydro-3-deoxygluconate/galactonate (2-dehydro-3-deoxygluconate/galactonate kinase) were found, the pathway seems to be non-phosphorilating as it was also previously shown for another *Thermoplasmatales* euryarchaeon *Picrophilus torridus* and discussed[1].

Non-phosphorylating pentosophosphate pathway involved in anabolic pentoses synthesis from fructose-6-phosphate and glyceraldehydes-3-phosphate was also encoded in both *Cunicliplasma*. The following enzymes were found (in S5 numeration): transketolase (CSP5_0843-0842), transaldolase (CSP5_0841), ribulose-phosphate 3-epimerase Rpe (CSP5_0267) and ribose-5-phosphate isomerase Rpi (CSP5_1191).

Based on the genomic annotation solely with non-phosphorylating ED and 5P pathways and non-operative glycolytic EM pathway it seems *Cuniculiplasma* cannot gain ATP from all these pathways and rely exclusively on fermentation of peptides or oxygen respiration.

Resistance to antibiotics and toxic compounds

Life in heavy metal environments has also a genomic reflection in a number of genes encoding for copper homeostasis counterparts, mercuric reductases, arsenic and cobalt-zinc-cadmium resistance. We thus think that based on the genomes annotation the archaeon S5 is more tightly equipped with heavy metal binding proteins such as a mercuric ion reductase (EC 1.16.1.1)/heavy metal reductase, occurred in four copies in contrast to scarce one copies in the PM4. Another difference between these organisms in this context reflected in pools of genes referred to two copies of YHS domain

copper/silver-binding protein and copper/silver-transporting P-type ATPase in S5 and only one copy in PM4.

Oxidative stress

The genomes of both organisms harbour three copies of hemerythrin HHE cation binding domain containing proteins (CPM_0233, CPM_0919, CPM_1919 and CSP5_0265, CSP5_0923, CSP5_1987). Hemerythrins are known to be rare in archaea, general functions of these proteins are referred to a binding of oxygen in mechanisms of delivery, sensory or detoxification in prokaryotic organisms or binding of iron or other metals in eukaryotes[4]. One could speculate on any of these functional strategies employed by these archaea, the clustering of the gene encoding peroxiredoxin family protein (CPM_1918 and CSP5_1986) may point at some detoxification potential. Other proteins to cope with stress, such as peroxiredoxins, peroxidases, thioredoxins, rubrerytrin and superoxide dismutase are represented across chromosomes of S5 and PM4.

Protein folding

Protein folding is considered to be important for all organisms and represents special importance for extreme acidophiles. Genes for AAA superfamily ATPase-domain containing proteins were identified in both genomes with four loci in PM4 and five in S5, PPases found to be FKBP-type peptidyl-prolyl cis-trans isomerase (homologous with genes from other *Thermoplasmatales*) and cyclophilin type peptidyl-prolyl cis-trans isomerase (revealed homology to methanogens counterparts). Similarly to the genome of *T. acidophilum*, genomes S5 and PM4 harbour gene clusters coding for DnaJ, DnaK and GrpE, a complete Hsp60 system (thermosome subunits α and β and prefoldins A

and B) and Hsp20 family proteins. Two genomes also harbour glutaredoxin-related protein/thiol-disulfide isomerase/thioredoxin.

Distribution patterns of arCOGs in Thermoplasmatales

The comparison of core genomes of *Cuniculiplasma* and all four other genera of *Thermoplasmatales* (apart from *Thermogymnomonas*) revealed relatively similar distribution patterns of arCOGs, but certain tendencies could be observed. Cell motility (FC="N") was characteristic for *Thermoplasma* and *Cuniculiplasma* only, despite the fact that microscopic images and genome mining did not reveal any flagellar apparatus in *Cuniculiplasma*[5]. Pili-associated hits were included into this category.

Another observation is related with a higher numbers of arCOGs of D, J, L, O, P, S, T, U and V categories, which may also be explained by the fact that *Cuniculiplasma* spp. possess largest genomes among *Thermoplasmatales* characterised so far (Fig. S4).

SUPPLEMENTARY FIGURES

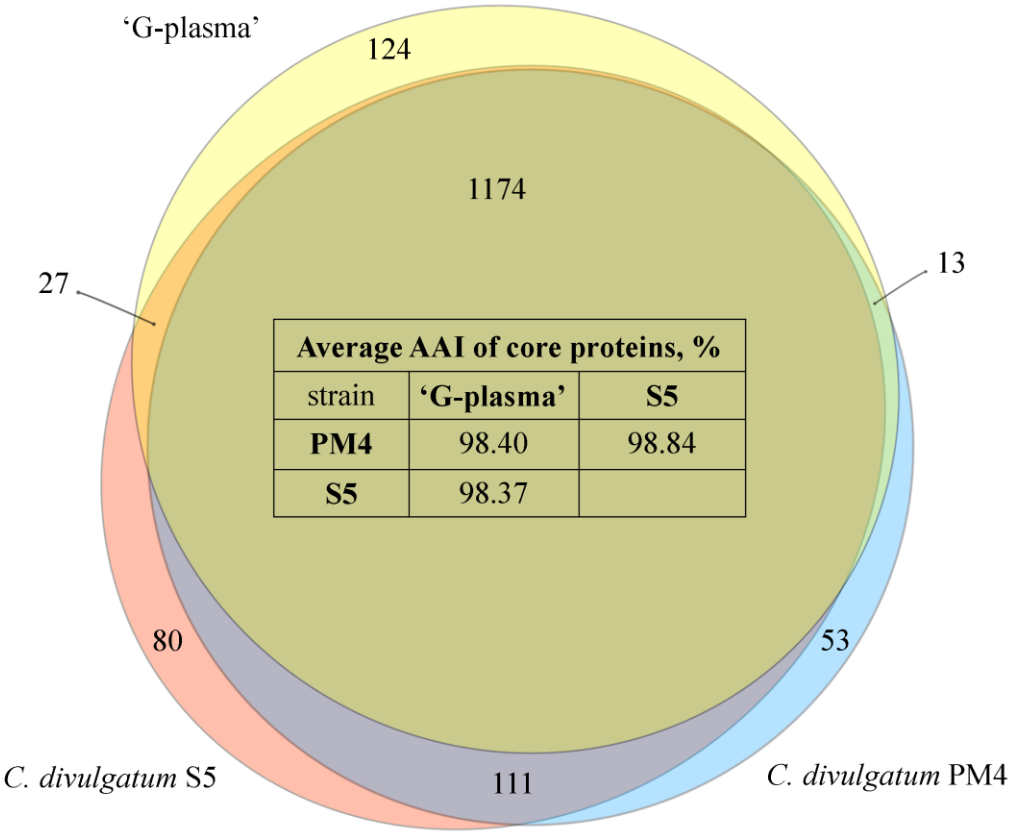

Supplementary Figure S1. Proportional Venn diagram on protein orthologs shared by the isolates. Orthology analysis of all high-confidence e *in silico* called proteins with length > 150 AA was performed with OrthoMCL suite using blastp e-value of 10^{-5} and grain value (used for orthology graph building) of 2.5[6]. Numbers indicate shared or unique protein clusters or singletons. Area of ellipses and their intersection is proportional to the number of proteins in each group. Diagram was drawn with eulerAPE software[7].

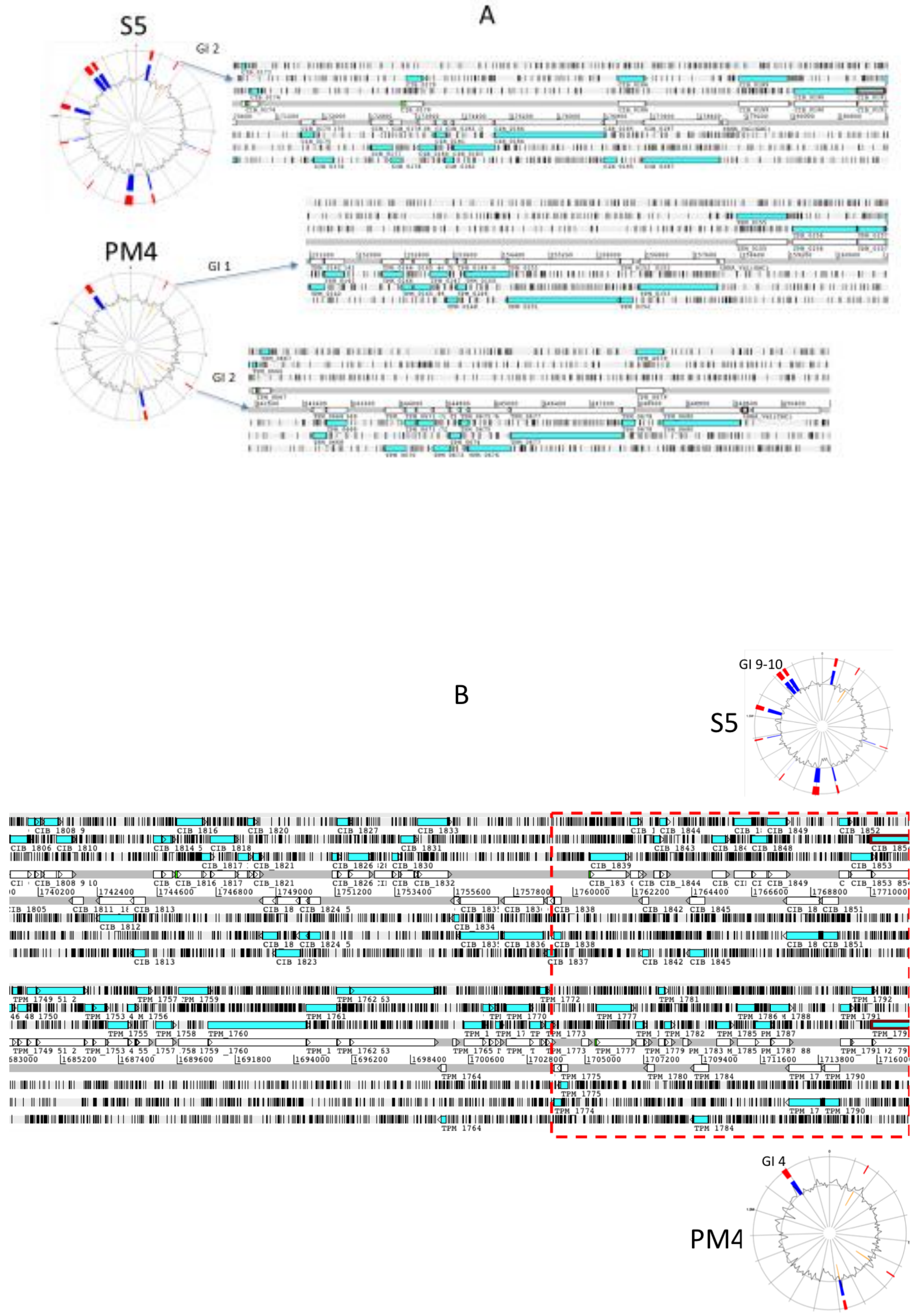

Supplementary Figure S2. Collinearity of gene clusters in genomic islands of *Cuniculiplasma divulgatum* PM and S5. **A.** Genomic islands 1 and 2 in strain PM4 both show identical gene arrangements with GI2 in strain S5; **B.** GI 9-1- in the strain S5 and GI4 in the strain PM4, similar regions highlighted by a box with dashed red line.

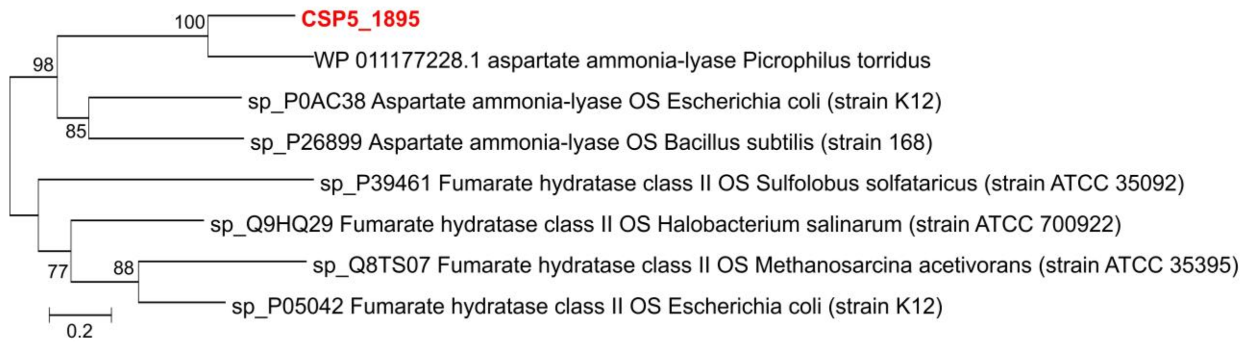

Supplementary Figure S3. Maximum Likelihood phylogenetic tree of CSP5_1895 (=CPM_1834) and several characterized homologous lyases and fumarate hydratases. The tree with the highest log likelihood (-6576.6741) is shown. The percentages of trees in which the associated taxa clustered together (bootstrap values, 100 replicates) are shown next to the branches. The tree is drawn to scale, with branch lengths measured in the number of substitutions per site. All positions with less than 95% site coverage were eliminated. There were a total of 424 positions in the final dataset. The tree was constructed in MEGA 6[8].

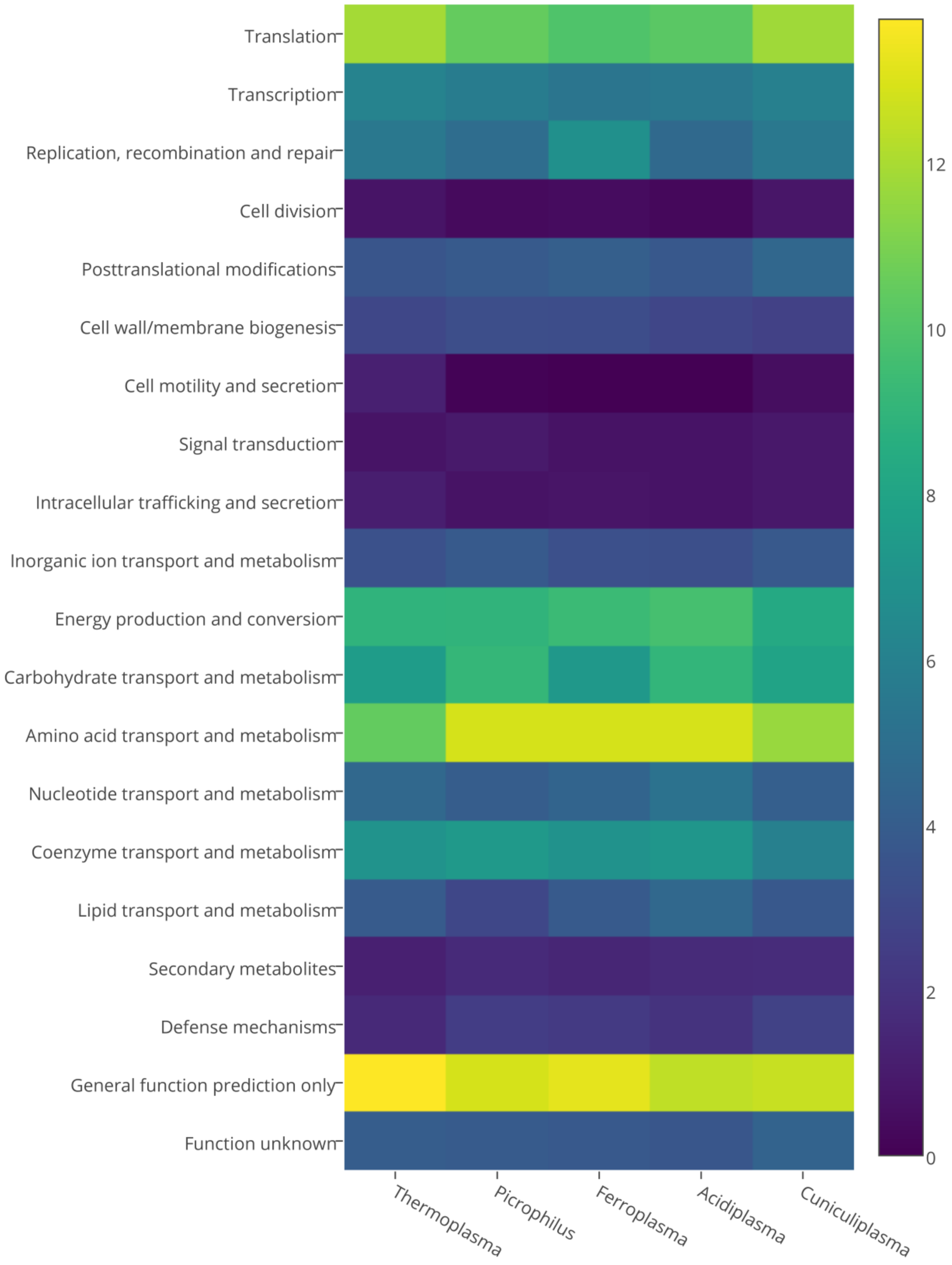

Supplementary Figure S4. Distribution of COGs functional categories among *Thermoplasmatales* representatives. For each genus, the core COGs of the type species were used for calculations.

SUPPLEMENTARY TABLES

Supplementary Table S1. Results of database mining for *Cuniculiplasma* - related 16S rRNA genes.

Database	Percent Identity	rRNA region	gene length in DB	Project name	Project (experiment) ID	Location	Lat	Lon
IMG Metagenome	100.00	full length (metagenome)	1469	Acid Mine Drainage (ARMAN) microbial communities from Richmond mine, Iron Mountain, CA, sample from Ultra Back A BS (re-annotation)	Gp0051182	Richmond mine, Iron Mountain, CA, USA	40.678	-122.515
	100.00	partial (metagenome)	1291	Pink biofilm microbial community from flowing acid mine drainage at Richmond mine, Iron Mountain, California, USA	Gp0097388	Richmond mine, Iron Mountain, CA, USA	40.678	-122.522
	94	partial (metagenome)	704	Acid mine drainage microbial communities from Los Rueldos abandoned mercury mine in Spain - Sample B2A	Gp0097859	Los Rueldos, Spain	43.25	5.767
	94	partial (metagenome)	1272	Acid mine drainage microbial communities from Los Rueldos abandoned Hg mine in Spain - Sample B1B	Gp0097858	Los Rueldos, Spain	43.25	5.767
	98	partial (metagenome)	827	Subaerial biofilm microbial communities from sulfidic caves, Italy, that are extremely acidic - Ramo Sulfureo RS9		Frasassi Cave System, Italy	43.40	12.96
	98	partial (metagenome)	796	Subaerial biofilm microbial communities from sulfidic caves, Italy, that are extremely acidic - Ramo Sulfureo RS9 (RS9 metagenome (Draft assembly w/ Newbler v. 2.6, contigs > 300 bp)		Frasassi Cave System, Italy	43.40	12.96
NCBI SRA archive	99	amplicon V3-V4	280	A microbial consortium enriched from acid mine drainage enhanced Cd phytoextraction	SRX1438201	China	missing	missing

				with minor effects on the indigenous soil microbial community				
	96	amplicon V3-V4	292	Acid Mine Drainage contaminated watershed Metagenome	SRX655594	Guiyang, China	26.60	106.63
	99	amplicon V3-V4	412	Copper Cliff Ontario Acid Mine Drainage	SRX326759	Copper Cliff, Ontario, Canada	48.46	-81.06
	96	full metagenome (not assembled)	NA	Meta_AMD_Lake<br>Meta_AMD_Flu	SRX079112<br>SRX079111<br>SRX079110	Yunfu mine, Guangdong, China	24.53	113.71
NCBI nt/nr	96	partial	861	Uncultured archaeon partial 16S rRNA gene, clone B133606F08	FN863008.1	Rio Tinto, Andalusia, Spain	37.59	6.55
	99	partial	1342	Uncultured archaeon gene for 16S rRNA, partial sequence, clone: HO28S9A20	AB600334.1	Kanagawa, Hakone, Ohwakudani, Japan	35.24	138.02
	99	partial	502	acidic biofilm from a pyrite mine in the Harz Mountains/Germany	KC127720.1	Harz Mountains/Germany	51.76	10.82
	96	partial	915	uncultured archaeon clone AMD-archD15 16S ribosomal RNA gene sequence	KC537525.1	Tongling mine, China	30.88	117.89
	99	partial	761	Uncultured archaeon clone NEC09069 16S ribosomal RNA gene, partial sequence	AY911458.1	Norris Geyser Basin, Yellowstone National Park, USA	44.73	-110.42
	100	full	1469	Uncultured Thermoplasmatales archaeon clone METASED06 16S ribosomal RNA gene, partial sequence	KJ907759.1	Michoacan, Los Azufres, Mexico	19.78	-100.64
	99	partial	833	Archaeon enrichment culture clone AA01 16S ribosomal RNA gene, partial sequence	KP676175.1	Cyprus	34.97	33.27

Supplementary Table S2. Overview of shared and unique proteomes of strains S5 and PM4 and ‘G-plasma’**.**

Gene group*	S5	PM4	G-Plasma
Single-copy genes, core	1145	1145	1145
Gene clusters, core, equal number of copies	17 (8)**	17 (8)	17 (8)
Gene clusters, core, differentially presented	28 (21)	31 (21)	40 (21)
Single copy genes. S5/G-Plasma	27	NA	27
Gene clusters. S5/G-Plasma	0 (0)	NA	0 (0)
Single copy genes. S5/PM4	109	109	NA
Gene clusters. S5/PM4	4 (2)	2 (2)	NA
Single copy genes. G-plasma/PM4	NA	13	13
Gene clusters. G-plasma/PM4	NA	0 (0)	0 (0)
Single copy genes. Strain-specific singletons	79	52	114
Gene clusters. Strain-specific.	11 (1)	2 (1)	21 (10)
Total proteins in OrthoMCL analysis	1420	1371	1377

*only genes coding proteins of length > 150 AA were included in the analysis
**number of protein clusters formed by protein group is shown in brackets

Supplementary Table S3. GC content and phylogenetic affiliations of encoded proteins in detected GIs.

	GI coordinates	GI length, bp	GC content, %	Taxonomic affiliation, phylum	Function
Cuniculiplasma divulgatum PM4					
GI_1	153207..157995	4789	38.9	*Euryarchaeota*	non-specific
GI_2	644304..649494	5391	38.1	*Euryarchaeota*	non-specific
GI_3	869564..881588	12025	39.6	*Euryarchaeota*	non-specific
GI_4	1680442..1702900	22459	41.2	*Euryarchaeota*	defense
GC-content of *C. divulgatum* PM4 genome without GIs			**37.1**	-	-
GC-content of total *C. divulgatum* PM4 genome			**37.2**	-	-
Cuniculiplasma divulgatum S5					
GI_1	58356..71416	13061	43.2	*Euryarchaeota*	non-specific
GI_2	173423..178764	5342	38.2	*Euryarchaeota*	non-specific
GI_3	585489..588931	3443	39.7	*Euryarchaeota*	defense
GI_4	891215..900387	9173	36.0	*Euryarchaeota*	transporters
GI_5	983534..1015555	32022	37.2	*Euryarchaeota*	transporters
GI_6	1174527..1179627	5101	39.3	*Euryarchaeota*	non-specific
GI_7	1385859..1392667	6809	38.4	*Euryarchaeota*	defense
GI_8	1531772.1552302	20531	37.7	*Euryarchaeota*	metals transport, efflux and redox
GI_9	1713043..1736229	23187	42.0	*Euryarchaeota*	defense
GI_10	1747587..1763197	15611	41.9	*Euryarchaeota*	defense
GC-content of *C. divulgatum* S5 genome without GIs			**37.1**	-	-
GC-content of total *C. divulgatum* S5 genome			**37.3**	-	-

Supplementary Table S4. S5 and PM4 Cas proteins show a similar degree of relatedness to counterparts from cultured *Archaea* and *Bacteria*.

	Top homologs in archaea*	e-value	Identity/ Similarity %	Top homologs bacteria	e-value	Identity/ Similarity%
**S5 (cluster CSP_1642-1637)**						
Cas3	EQB68015 G-plasma	0.0	100/100	WP_008871467 Desulfonatronospira thiodismutans	0.0	41/60
	CCJ37384 Methanoculleus bourghensis	0.0	43/62	WP_015752049 Desulfohalobium retbaense	0.0	40/58
Csx17	EQB68016 G-plasma	0.0	100/100	WP_015752050 Desulfohalobium retbaense	2e-139	33/54
	WP_011308049 Methanosarcina barkeri	4e-83	28/46	WP_008871466 Desulfonatronospira thiodismutans	2e-123	32/52
Cas7	EQB68017 G-plasma	0.0	100/100	WP_015752051 Desulfohalobium retbaense	5e-123	55/76
	WP_011308048 Methanosarcina barkeri	7e-79	44/62	WP_008871467 Desulfonatronospira thiodismutans	5e-115	51/71
Cas5	EQB68017 G-plasma	0.0	100/100	WP_008871464 Desulfonatronospira thiodismutans	5e-104	38/56
	WP_011308048 Methanosarcina barkeri	5e-55	28/45	WP_015752052 Desulfohalobium retbaense	5e-100	36/56
Cas4/Cas1	EQB66602 E-plasma	0.0	54/71	WP_041975875 Pyrinomonas methylaliphatogenes	0.0	49/66
	WP_014587242 Methanosaeta harundinacea	33e-148	44/60	WP_044936209 Acidobacterium ailaaui	0.0	48/65
Cas2	EQB68021 G-plasma	3e-61	100/100	WP_060635480 Pyrinomonas methylaliphatogenes	2e-26	46/71
	WP_014587243 Methanosaeta harundinacea	2e-22	43/64	WP_014101551 Chloracidobacterium thermophilum	1e-20	40/69

PM4 (cluster CPM_1611-1604)						
Cas6	WP_009887248 “Ferroplasma acidarmanus”	7e-54	41/60	WP_035163074 Caloranaerobacter azorensis	6e-50	38/50
	WP_054839294 Sulfolobus metallicus	7e-37	30/52	WP_025748599 Caldicoprobacter oshimai	6e-49	37/60
Cas8b	WP_009887249 “Ferroplasma acidarmanus”	4e-57	28/48	WP_052217992 Thermincola ferriacetica	4e-47	28/48
	WP_015286123 Methanoregula formicica	2e-40	27/44	AEB12791 Marinithermus hydrothermalis DSM 14884	4e-44	25/44
Cas7	WP_009887251“Ferroplasma acidarmanus”	8e-39	40/57	WP_051321975 Alicyclobacillus contaminans	1e-42	35/60
	SCG85879 Methanobacterium curvum	3e-23	28/50	WP_067929302 Alicyclobacillus shizuokensis		34/55
Cas5	WP_009887252 “Ferroplasma acidarmanus”	1e-170	37/57	WP_067933424 Alicyclobacillus kakegawensis	3e-139	33/57
	WP_015286120 Methanoregula formicica	2e-117	34/55	WP_067929303 Alicyclobacillus shizuokensis	4e-139	33/56
Cas3	WP_009887252 “Ferroplasma acidarmanus”	1e-168	37/57	WP_067933424 Alicyclobacillus kakegawensis	4e-139	33/57
	WP_015286120 Methanoregula formicica	2e-117	34/55	WP_067929303 Alicyclobacillus shizuokensis	7e-139	33/56
Cas4	WP_014733242 Pyrococcus sp. ST04	9e-38	44/61	WP_012033071 Pelotomaculum thermopropionicum	7e-53	47/68
	WP_013906184 Pyrococcus yayanosii	8e-35	39/61	SDY05938 Eubacterium barkeri	2e-52	50/67
Cas1	WP_009887254 “Ferroplasma acidarmanus”<br><br>WP_015590840 Archaeoglobus sulfaticallidus	1e-125<br><br>4e-93	51/74<br><br>44/64	WP_066353963 Fervidicola ferrireducens<br><br>SDW90767 Tepidimicrobium xylanilyticum	1e-128<br><br>9e-126	54/73<br><br>53/77

Cas2	WP_019841618<br>“Ferroplasma acidarmanus”	8e-28	57/74	WP_024833242<br>Clostridium josui	1e-31	63/77
	WP_054839124<br>Sulfolobus metallicus	2e-22	53/66	WP_011876923<br>Desulfotomaculum reducens	3e-29	59/74

*top homolog from nrNCBI followed by the top hit from archaea with validly published names

Supplementary Table S5. CRISPRs in the genomes of *Cuniculiplasma divulgatum* S5 and PM4.

See a separate Microsoft Excel file Supplementary Table S5.

Supplementary Table S6. Genomic islands (GIs) in the genomes of *Cuniculiplasma divulgatum* S5 and PM4.
GIs were predicted using Islandviewer 3[9]. Marked are the proteins potentially involved in transport, oxidative stress response and metal efflux (turquoise), and horizontal gene transfer ‘defense’ systems: DNA repair, restriction-modification and toxin-antitoxin systems (yellow), with their corresponding top psi-blast hits to proteins within *Thermoplasmata* (cut-off at the e-value of 0.005), Aae – *Acidiplasma aeolicum*, Acu – *Acidiplasma cupricumuans*, Fac – ‘*Ferroplasma acidarmanus*’, Pto – *Picrophilus torridus*, Tac – *Thermoplasma acidophilum*, Tvo – *Thermoplasma volcanium*; Apl, Epl, Gpl, Ipl correspond to ‘alphabet plasmas’ metagenomic assemblies. Clusters of genes having the same arrangements in GIs in both isolates (S5 GI9-10 vs PM4 GI4 and S5 GI2 vs PM4 GI2 and PM4 GI3) are marked in pink, tRNAs are marked in red.

See a Separate Microsoft Excel file Supplementary Table S6.

SUPPLEMENTARY INFORMATION REFERENCES

1. Bräsen, C., Esser, D., Rauch, B. & Siebers, B. Carbohydrate metabolism in Archaea: current insights into unusual enzymes and pathways and their regulation. *Microbiol Mol Biol Rev.* **78,** 89-175 (2014).
2. Fütterer, O. *et al*. Genome sequence of *Picrophilus torridus* and its implications for life around pH 0. *Proc Natl Acad Sci USA* **101(24),** 9091-9096 (2004).
3. Reher, M., Fuhrer, T., Bott, M. & Schönheit, P. The nonphosphorylative Entner-Doudoroff pathway in the thermoacidophilic euryarchaeon *Picrophilus torridus* involves a novel 2-keto-3-deoxygluconate-specific aldolase. *J. Bacteriol*. **192,** 964-974 (2010).
4. French, C. E., Bell, J. M. & Ward, F. B. Diversity and distribution of hemerythrin-like proteins in prokaryotes. *FEMS Microbiol. Lett*. **279,** 131-145 (2008).
5. Golyshina, O. V. *et al*. The novel, extremely acidophilic, cell wall-deficient archaeon *Cuniculiplasma divulgatum* gen. nov., sp. nov. represents a new Family of *Cuniculiplasmataceae* fam. nov., *order Thermoplasmatales. Int. J. Syst. Evol. Microbiol.* **66,** 332-340 (2016).
6. Li, L., Stoeckert, C. J. Jr. & Roos D. S. OrthoMCL: identification of ortholog groups for eukaryotic genomes. *Genome Res.* **13,** 2178-2189 (2003).
7. Micallef, L. & Rodgers, P. eulerAPE: drawing area-proportional 3-Venn diagrams using ellipses. *PLoS One* **9**, e101717 (2014).
8. Tamura, K., Stecher, G., Peterson, D., Filipski, A. & Kumar, S. MEGA6: Molecular Evolutionary Genetics Analysis version 6.0. *Mol. Biol. Evol.* **30**, 2725-2729 (2013).
9. Dhillon, B.K. *et al*. IslandViewer 3: more flexible, interactive genomic island discovery, visualization and analysis. *Nucl. Acids Res.* **43,** W104-W108 (2015).
